# Impact of Osteoporosis in Postmenopausal Women With Primary Knee Osteoarthritis

**DOI:** 10.7759/cureus.40645

**Published:** 2023-06-19

**Authors:** Mohammed Zamzam, Muhannad S Alamri, Fayez G Aldarsouni, Homoud Al Zaid, Abdulhadi A Al Ofair

**Affiliations:** 1 Department of Orthopedics, College of Medicine, King Saud University, Riyadh, SAU; 2 Department of Internal Medicine, College of Medicine, King Saud University, Riyadh, SAU; 3 Department of Surgery, College of Medicine, King Saud University, Riyadh, SAU; 4 Department of Pharmacy, King Saud University, Riyadh, SAU

**Keywords:** t-score, osteoporosis, osteoarthritis, bone mineral density, bone mass index

## Abstract

Background: Although knee osteoarthritis (OA) and osteoporosis (OP) are common age-related bone disorders, the association between the two conditions remains indefinable. The aim of the present study is to investigate a possible relationship between the two conditions in post-menopausal women.

Methodology: A cross-sectional study was conducted at King Khalid University Hospital, Riyadh, Saudi Arabia, after obtaining IRB approval. The study included all post-menopausal female patients above 50 years of age, who underwent dual-energy X-ray absorptiometry (DXA) scans between January 2019 and July 2021 and have clear radiological data regarding knee OA. For our study, 487 ladies satisfied our inclusion criteria with an age range of 50-94 years (mean 64.67 ±8.4). The severity of knee OA was determined according to the Kellgren and Lawrence (KL) grading system.

Results: The mean age and weight of all patients showed a significant association with knee OA. There was no significant association between bone quality and all parameters of bone mineral density (BMD) and T-score with the presence of knee OA. The mean age, height, weight, and BMI have a significant relationship with OP. Grade 3 of the KL classification system for knee OA is the only grade that showed a significant relationship with the presence of OP. The status of bone quality, BMD, and T-score parameters have no significant relationship with the severity subgrouping of knee OA. The BMD and the T-score of the right femoral neck have a significant correlation with BMI, which is linked to the presence of knee OA.

Conclusion: We didn’t find a clear relationship between OP or BMD and the presence of knee OA. However, our findings demonstrated that BMD, T-score of the right femoral neck, and BMI can all be utilized as predictors for the development and progression of knee OA. We recommend considering the many potential confounding variations when describing a relationship between OP and OA.

## Introduction

Knee osteoarthritis (OA) is a joint condition prevalent among the aged, characterized by degenerative changes in various joint components, including bone and cartilage [1]. Low bone mineral density (BMD) and micro-architectural degeneration of the bone define osteoporosis (OP), a common metabolic disease linked with age. Although OA and OP are distinct diseases, they have common risk factors and bone metabolism derangements. While certain risk factors, such as age and gender, are convergent, others, such as body mass index (BMI) and physical activity, are divergent [2]. Despite knee OA and OP being common age-related bone disorders, the association between the two conditions remains elusive, and no conclusive link has been established [3]. Many studies have been conducted to establish a definitive link between the two conditions [4]. However, the findings were inconsistent and varied according to several factors, which include the severity of OA, parameters used to define OA, study design, and the site affected by osteoarthritic changes.

When OA affects the knees, it has been found that its incidence is associated with increased axial BMD. On the other hand, when OA affects the hand, its incidence is not associated with increased axial BMD [5]. These findings demonstrate how the relationship between the two diseases differs based on the site. When utilizing the Kellgren-Lawrence (KL) grading system for knee OA [6], the incidence was positively correlated with increased axial BMD [7]. However, when OA is defined by joint space narrowing (JSN), the association between axial BMD and the incidence of knee OA doesn’t exist [8].

Moreover, in another study, the severity of OA as classified by the KL grading system alters the relationship between OA and OP. There has been a steady increase in the T-score of the hip and lumbar spine in a linear fashion with the increase in the KL grading system from 0 to 2. But moderate to severe OA (grades 3 and 4) are associated with decreased T-score [9].

A definitive link between the two diseases has not been elucidated. Nevertheless, numerous benefits are expected if a relationship between the two conditions can be confirmed. Once a clear link has been established, the risk of developing OP is expected whenever a patient is diagnosed with OA, and thus preventive measures could be taken and vice versa. The present study aims to shed light on this dilemma by investigating a possible relationship between the two conditions and trying to find a link between OA and OP in post-menopausal women by evaluating their bone quality (through calculating their BMD and T-score in both lumbar spine and femoral neck), BMI and the severity of knee OA according to KL grading system.

## Materials and methods

After obtaining IRB approval, a cross-sectional study was conducted at King Khalid University Hospital, Riyadh, Saudi Arabia. Data were collected from all patients who presented at the Nuclear Medicine department for measuring bone density between January 2019 and July 2021. The collected data were obtained from medical records through Electronic System for Integrated Health Information Software (E-SIHI) used in the authors' institute.

The study included all postmenopausal female patients ≥ 50 years of age, who underwent dual-energy X-ray absorptiometry (DXA) scan and had precise radiological data regarding knee OA. Exclusion criteria were those ladies who were under 50 years of age and diagnosed from another institute; also those who had secondary knee OA or insufficient data.

Of 1836 patients, only 487 ladies satisfied our inclusion criteria with an age range from 50-94 years (mean, 64.67 ±8.4). All participants were categorized based on age into four categories: 50-59, 60-69, 70-79, and ≥80 years of age. According to their BMI, patients were classified as well based on World Health Organization (WHO) categories of BMI into underweight (BMI less than 18.5), normal (BMI between 18.5 and 24.9), overweight (BMI between 25 and 29.9), obese (BMI between 30 and 35), and morbidly obese (BMI more than 35). 

Patients were divided into two groups, 141 with knee OA (group I) and 346 without knee OA (group II). The diagnosis of OP was established according to Lunar iDXA (GE HealthCare Technologies Inc., Chicago, IL). OP is defined as lumbar spine (L1-L4) or femoral neck BMD and T-score ≥ 2.5 below standard deviations (SD) of the young-adult reference mean of both parameters. Osteopenia is defined as BMD and T-score of 1.0 to 2.5 below SD. BMD and T-score within 1.0 below SD were considered normal. There were 206 patients with OP, 209 with osteopenia, and 72 normal.

Diagnosis of knee OA was established upon the presence of its radiological features, and the severity was determined according to the KL grading system. In his system, grade 0 has no JSN or reactive changes. Grade 1 has doubtful JSN and possible osteophyte lipping. Grade 2 has definite JSN and possible osteophytes. Grade 3 has moderate osteophytes, definite JSN, some sclerosis, and possible bone-end deformity. Grade 4 has large osteophytes, marked JSN, severe sclerosis, and solid bone ends deformity. Patients who underwent total knee replacement (TKR) were considered Grade 4. 

For data analysis, we used SPSS 26 version (IBM SPSS Inc. Armonk, NY). Values were reported using means (SD) for continuous variables or n (%) for categorical variables. P-values were found by analysis of variance, independent t-test for continuous variables and the chi-square test, Fisher's exact test, and Monte-Carlo exact test for categorical variables. Mann-Whitney U test, linear regression, correlation analysis, and the chi-squared test were used to calculate the p-value. If p<0.05, the result was considered significant.

## Results

Table 1 shows the distribution of the presence of knee OA in obese (BMI ≥30) and non-obese (BMI <30) patients according to different age groups.

**Table 1:** Distribution of knee OA in obese (BMI>30) and non-obese (BMI<30) patients according to different age groups Data are presented as numbers (N) and percentages (%)

Parameter	Total (%)	No Knee OA (%)			With Knee OA (%)	
	(N=487) (100)	(N=346) (71)			(N=141) (29)	
BMI						
BMI <30		212(61.3)			57(40.5)	
BMI ≥30		134(38.7)			84(59.5)	
Age in years						
50-59	130(26.7)	61(28.8)		40(29.9)	12(21.1)	17(20.2)
60-69	226(46.4)	87(41)	61(45.5)		32(56.1)	46(54.8)
70-79	97(19.9)	44(20.8)	27(20.1)		7(12.3)	19(22.6)
>80	34(7)	20(9.4)	6(4.5)		6(10.5)	2(2.4)

The mean age of all patients and those who are 50-59 and 60-69 years of age showed significant association with knee OA (p=0.021, 0.032, and 0.008 respectively). However, there was no significant relationship between height and mean BMI with the presence of knee OA (Table 2).

**Table 2:** Demographic and anthropometric characteristics of all patients in relation to diagnosis of knee OA Values are the means (SD) or n (%). * p-values were calculated by independent t-test for continuous variables and the chi-square test, Fisher's exact test was used to calculate categorical variables

Parameters	Total (%)	No Knee OA (%)	With Knee OA (%)	*p-value
	(N=487) (100)	(N=346) (71)	(N=141) (29)	
Married, Yes	410(84.2)	286(82.7)	124(87.9)	0.093
Age in Years	64.67	64.56	64.95	0.021
Mean (SD)	8.4	8.7	7.6	
Age 50-59	130(26.7)	101(29.2)	29(20.6)	0.032
Age 60-69	226(46.4)	148(42.8)	78(55.3)	0.008
Age 70-79	97(19.9)	71(20.5)	26(18.4)	0.349
Age more than 80	34(7)	26(7.5)	8(5.7)	0.306
Height, mean (SD)	1.57(0.145)	1.57(0.16)	1.56(0.05)	0.529
Weight, mean (SD)	73.779(`4.9)	72(13.9)	77.9(16.467)	0.014
BMI mean (SD)	30.082(5.88)	29.3(5.6)	31.96(6.1)	0.101
Underweight	6(1.2)	5(1.4)	1(0.7)	0.442
Normal	82(16.8)	65(18.8)	17(12.1)	0.045
Overweight	179(36.8)	142(41)	37(26.2)	0.001
Obese	124(25.5)	80(23.1)	44(35.5)	0.042
Morbidly obese	93(19.1)	53(15.3)	40(28.4)	0.001
Bone quality normal	72(14.8)	50(14.5)	22(15.6)	0.422
Osteopenia	209(42.9)	145(69.4)	64(45.4)	0.273
Osteoporosis	206(42.3)	151(43.6)	55(39)	0.201
BMD of lumbar spine, mean (SD)	0.946(0.15)	0.94(0.15)	0.95(0.14)	0.29
T-score of lumbar spine, mean (SD)	-1.98(1.227)	-2(1.25)	-1.9(1.149)	0.228
BMD of right femoral neck, mean (SD)	0.889(0.2)	0.88(0.23)	0.89(0.14)	0.36
BMD of left femoral neck, mean (SD)	2.7(40.4)	0.346(47.9)	0.9(0.1553)	0.212
T-score right femoral neck, mean (SD)	-0.927(1.6)	-0.926(1.78)	-0.929(1.17)	0.428
T-score left femoral neck, mean (SD)	-0.898(1.16)	-0.94(1.11)	-0.78(1.26)	0.255

A substantially greater weight (p=0.014) was seen in the females with knee OA. Further analysis of the relationship between weight and the presence of knee OA according to BMI subgrouping showed that patients with average weight or obesity had significant links (p=0.045 and 0.042, respectively). However, overweight patients or those who have morbid obesity had a stronger association (p=0.001) (Table 2).

There was no significant relationship between bone quality and knee OA. Table 2 showed that all parameters of BMD and T-score for the right and left femoral neck and lumber spine had no significant association with the presence of knee OA.

Table 3 shows all patients' demographic and anthropometric characteristics and the status of their bone quality according to BMD and T-score parameters. The mean age and age categories of 50-59 and ≥80 years have significant relation with OP (p=0.0002, 0.005, and 0.014, respectively). The same is the mean height (p=0.019), mean weight (p=0.000), mean BMI (p=0.0004), and BMI Categories of normal, overweight, and morbidly obese (p=0.048, 0.048, and 0.005, respectively). Grade 3 of the KL classification system for knee OA is the only grade with significant relation with OP (p=0.03).

**Table 3:** Demographic and anthropometric characteristics of all patients in relation to status of their bone quality Values are the means (SD) or n (%). * p-values were calculated by independent t-test for continuous variables and the chi-square test, Fisher's exact test was used to calculate categorical variables

Parameter	Total (%)	Normal (%)	*p-value	Osteopenia	*p-value	Osteoporosis	*p-value
	(N=487) (100)	72(14.8)		(N=209) (42.9)		(N=206) (42.3)	
Married, Yes	410(84.2)	60(83.3)	0.861	180(86.1)	0.06	170(82.5)	0.23
Age in years, mean (SD)	64.67(8.4)	60.5(6.9)	0.000007	64.4(8.1)	0.6	66.339(8.6)	0.0002
Age 50-59	130(26.7)	31(43.1)	0.001	58(27.8)	0.36	41(19.9)	0.005
Age 60-69	226(46.4)	32(44.4)	0.409	98(46.9)	0.46	96(46.6)	0.51
Age 70-79	97(19.9)	8(11.1)	0.026	41(19.6)	0.49	48(23.3)	0.07
Age more than 80	34(7)	1(1.4)	0.026	12(5.7)	0.23	21(10.2)	0.014
Height (m), mean (SD)	1.57(0.145)	1.6(0.35)	0.061010	1.56(0.05)	0.48	1.5(0.05)	0.019
Weight, mean (SD)	73.779(4.9)	80.9(13.7)	0.000010	75.2(14.8)	0.07	69.8(14.3)	0
BMI, mean (SD)	30.082(5.88)	31.4(6.2)	0.035	30.7(5.78)	0.04	28.9(5.6)	0.000386
Underweight	6(1.2)	1(0.2)	0.619	0(0)	0.034	5(2.4)	0.052
Normal	82(16.8)	7(9.7)	0.052	33(15.8)	0.34	42(20.4)	0.048
Overweight	179(36.8)	22(30.6)	0.147	72(34.4)	0.21	85(41.3)	0.048
Obese	124(25.5)	23(31.9)	0.112	56(26.8)	0.32	45(21.8)	0.07
Morbid obese	93(19.1)	19(26.4)	0.065	46(22)	0.097	28(13.6)	0.005
KL grades of knee OA, Grade 0	368(75.6)	52(73.2)	0.28	155(74.2)	0.30	161(78.2)	0.15
Grade 1	9(1.8)	1(1.4)	0.6	4(1.9)	0.51	4(1.9)	0.57
Grade 2	17(3.5)	4(5.6)	0.23	7(3.3)	0.54	6(2.9)	0.37
Grade 3	29(6)	5(6.9)	0.43	17(8.1)	0.06	7(3.4)	0.03
Grade 4	64(13.1)	10(13.9)	0.48	26(12.4)	0.399	28(13.6)	0.45

Table 4 shows the characteristics of all patients and their bone quality status according to the severity of knee OA if present. The mean age and age categories of 50-59 and 60-69 significantly correlate with the severity of knee OA (p=0.002, 0.002, and 0.015, respectively). The mean weight, average weight, and overweight (p=0.024, 0.015, and 0.0001, respectively). However, morbid obesity has a strong association (p=0.000). Nevertheless, bone quality, BMD, and T-score parameters have no significant relationship with the severity subgrouping of knee OA.

**Table 4:** Characteristics of all patients according to KL classification system for radiographic severity of knee OA Values are the means (SD) or n (%).*P values by analysis of variance for continuous variables, the chi-square test, Fisher's exact test was used for for categorical binary variables, and the Monte-Carlo exact test was used for multinomial variables

KL Grades	Total (N=487)	0 (n=374)	1 (n=9)	2 (n=17)	3 (n=29)	4 (n=64)	*p-value
Age in years, mean (SD)	64.6(8.4)	64.4(8.59)	61.6(6.2)	62.5(5.58)	64.32(5.5)	67.23(8.4)	0.002
Age 50-59	130(26.7)	110(29.9)	2(22.2)	4(23.5)	3(10.3)	11(17.2)	0.002
Age 60-69	226(46.4)	157(42.7)	6(66.7)	11(64.7)	22(75.9)	30(46.9)	0.015
Age 70-79	97(19.9)	76(20.7)	1(11.1)	2(11.8)	4(13.8)	14(21.9)	0.404
Age more than 80	34(7)	25(6.8)	0(0)	0(0)	0(0)	9(14.1)	0.154
Height, mean (SD)	1.57(0.14)	1.57(0.16)	1.58(0.03)	1.56(0.03)	1.56(0.05)	1.55(0.05)	0.977
Weight, mean (SD)	73.779(14.9)	72.03(13.8)	75.6(14.9)	70.7(11.8)	79.793(14.6)	81.6(18.7)	0.024
BMI, mean (SD)	30.08(5.8)	29.3(5.5)	29.9(5.8)	29.6(4.1)	32.75(5.9)	33.47(6.7)	0.183
Normal (18.5-25)	82(16.8)	70(19)	2(22.2)	1(5.9)	1(3.4)	8(12.5)	0.015
Overweight (25-30)	179(36.8)	149(40.5)	3(33.3)	8(47.1)	9(31)	10(15.6)	0.0001
Obese (30-35)	124(25.5)	87(23.6)	2(22.2)	6(35.3)	12(41.4)	17(26.6)	0.087
Morbid obese (>35)	91(19.1)	56(15.2)	2(22.2)	1(5.9)	6(20.7)	28(43.8)	0
Bone quality normal	72(14.8)	52(14.1)	1(11.1)	4(23.5)	5(17.2)	10(15.6)	0.278
Osteopenia	209(42.9)	155(42.1)	4(44.4)	7(41.2)	17(58.6)	26(40.6)	0.33
Osteoporosis	206(42.3)	161(43.8)	6(35.3)	7(24.1)	28(43.8)	28(43.8)	0.336
BMD of lumbar spine, mean (SD)	0.94(0.15)	0.94(0.15)	0.96(0.099)	0.94(0.16)	0.98(0.146)	0.94(0.14)	0.752
T-score of lumbar spine, mean (SD)	-1.9(1.22)	-2.01(1.2)	-1.8(0.79)	-2.03(1.3)	-1.6(1.2)	-1.9(1.17)	0.728
BMD of Right femoral neck, mean (SD)	0.89(0.21)	0.88(0.22)	0.87(0.07)	0.91(0.13)	0.93(0.14)	0.87(0.16)	0.735
BMD of left femoral neck, mean (SD)	2.7(40.4)	3.3(46.5)	0.88(0.127)	0.93(0.125)	0.96(0.151)	0.87(0.16)	0.873
T-score right femoral neck, mean (SD)	-0.927(1.6)	-0.93(1.7)	-1.078(0.6)	-0.729(1.04)	-0.629(1.1)	-1.081(1.29)	0.762
T-score left femoral neck, mean (SD)	-0.89(1.16)	-0.94(1.1)	-0.96(1.02)	-0.3(1.056)	-0.3(1.2)	-1.01(1.3)	0.507

**Figure 1:**
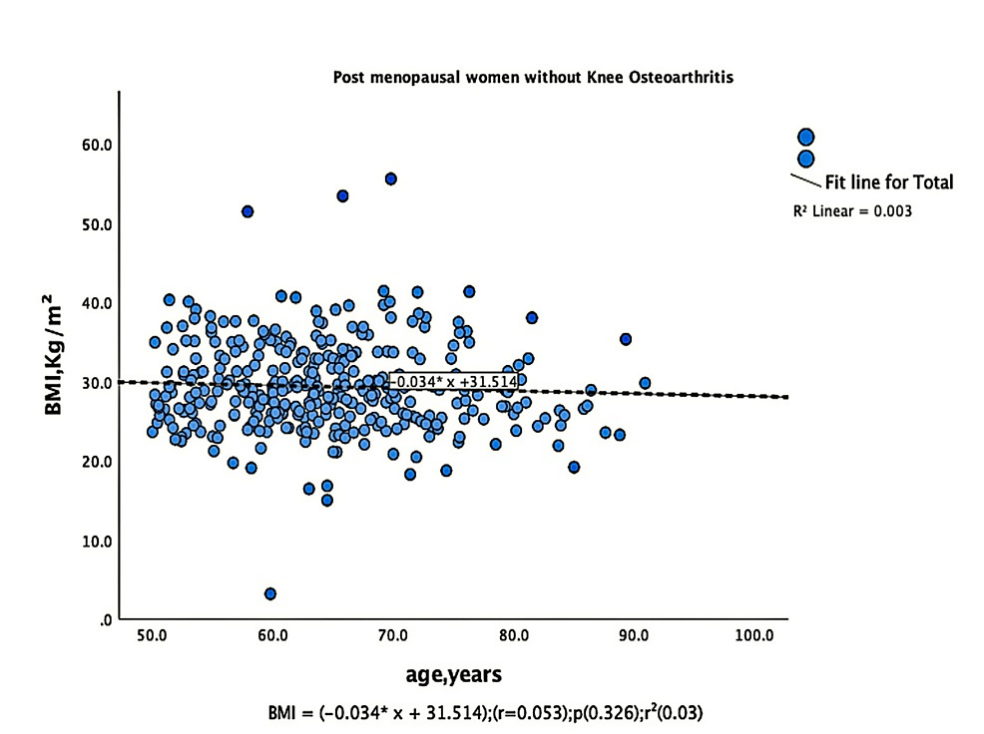
Correlation and regression analyses of relations between BMI (Y-axis) and age (X-axis) of post-menopausal women without osteoarthritis BMI: body mass index

**Figure 2:**
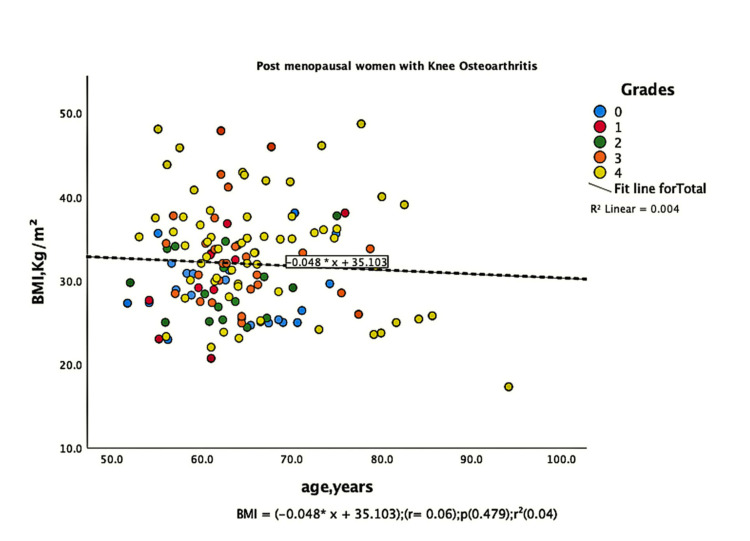
Correlation and regression analyses of relations between BMI (Y-axis) and age (X-axis) of post-menopausal women with osteoarthritis. BMI: body mass index

**Figure 3:**
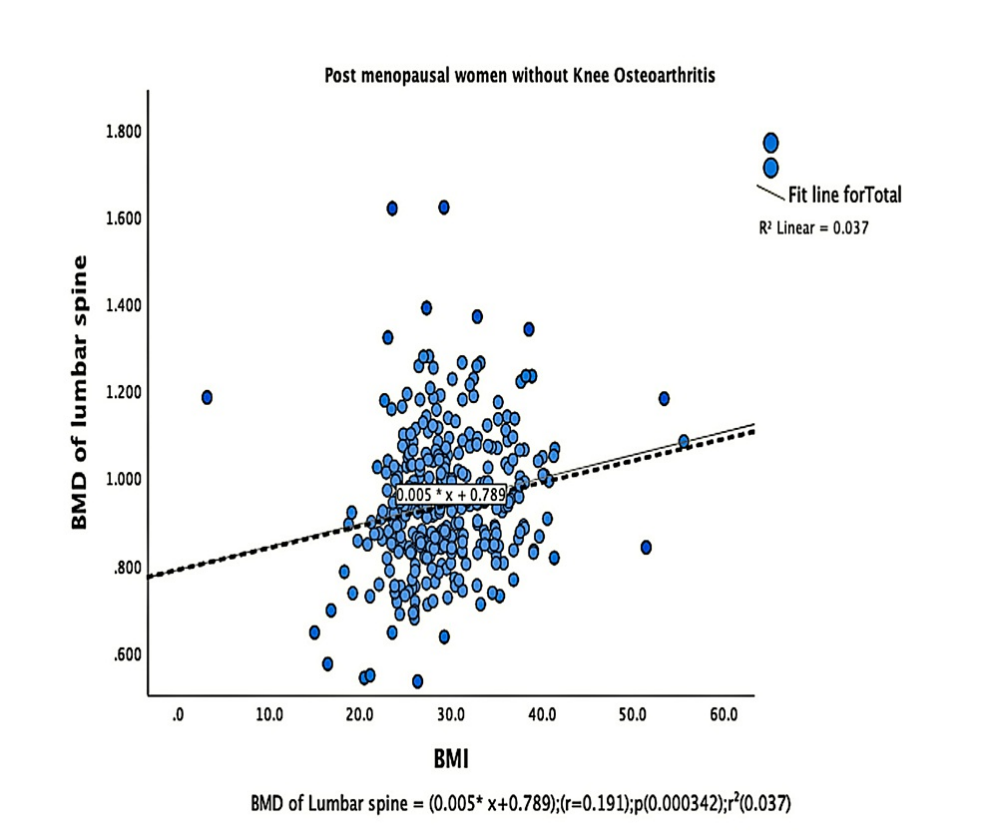
Correlation and regression analyses of relations between BMD of lumbar spine (Y-axis) and BMI (X-axis) of post-menopausal women without osteoarthritis. BMD: bone mineral density; BMI: body mass index

**Figure 4:**
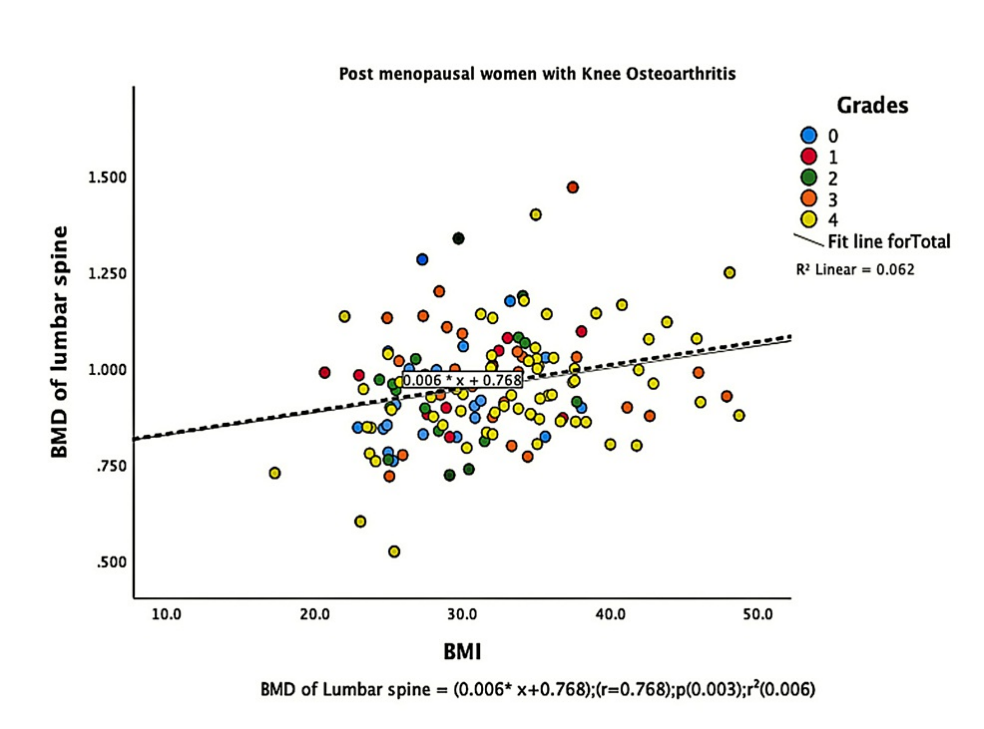
Correlation and regression analyses of relations between BMD of lumbar spine (Y-axis) and BMI (X-axis) of post-menopausal women with osteoarthritis. BMD: bone mineral density; BMI: body mass index

**Figure 5:**
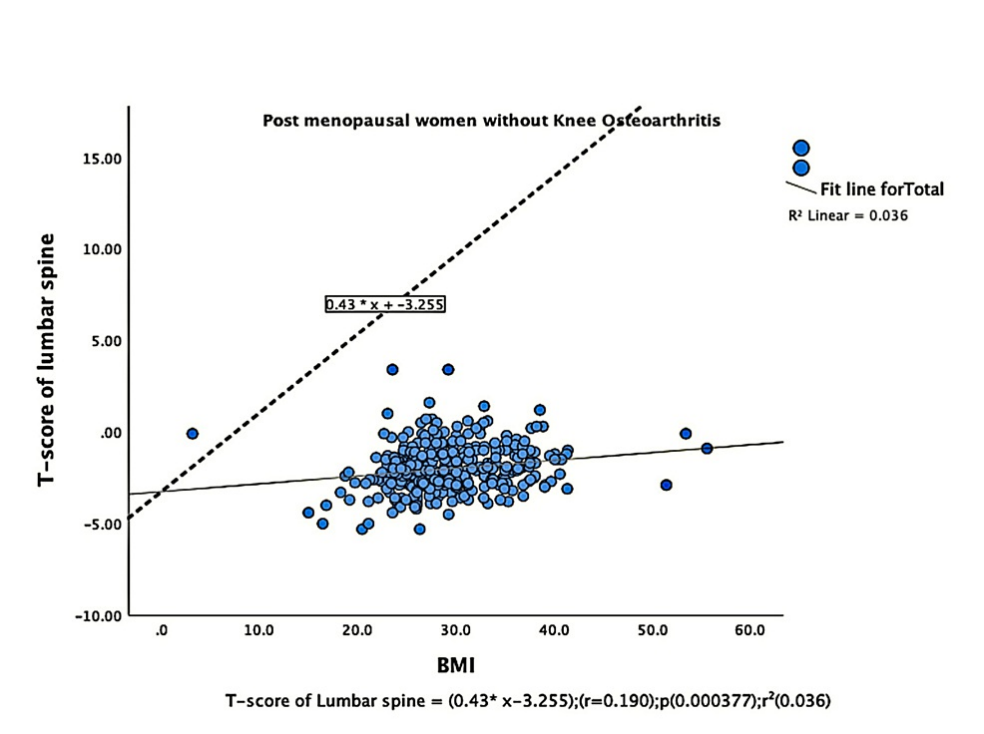
Correlation and regression analyses of relations between T-score of lumbar spine (Y-axis) and BMI (X-axis) of post-menopausal women without osteoarthritis BMI: body mass index

**Figure 6:**
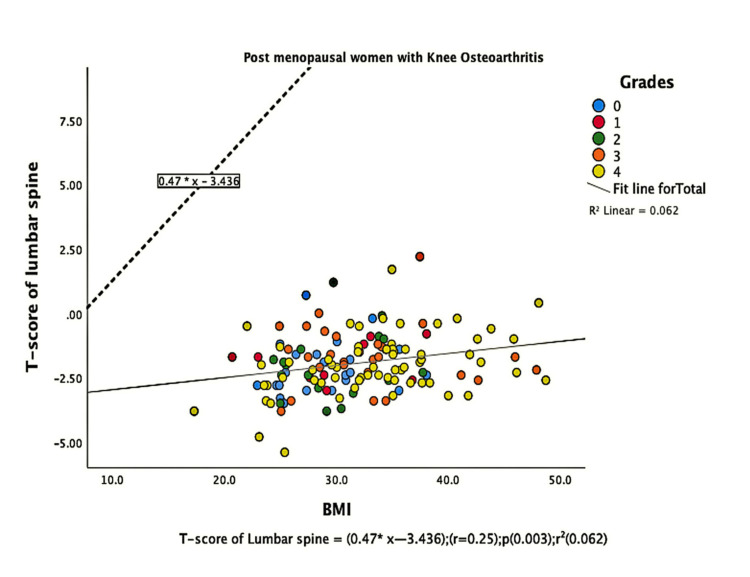
Correlation and regression analyses of relations between T-score of lumbar spine (Y-axis) and BMI (X-axis) of post-menopausal women with osteoarthritis. BMI: body mass index

**Figure 7:**
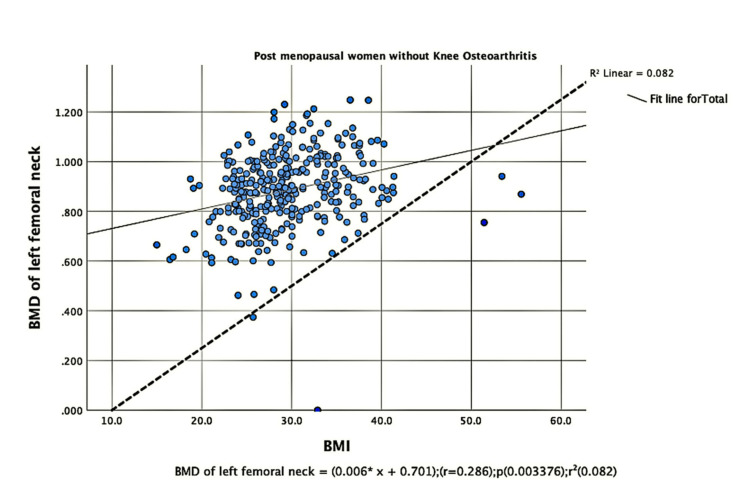
Correlation and regression analyses of relations between BMD of the left femoral neck (Y-axis) and BMI (X-axis) of post-menopausal women without osteoarthritis BMD: bone mineral density; BMI: body mass index

**Figure 8:**
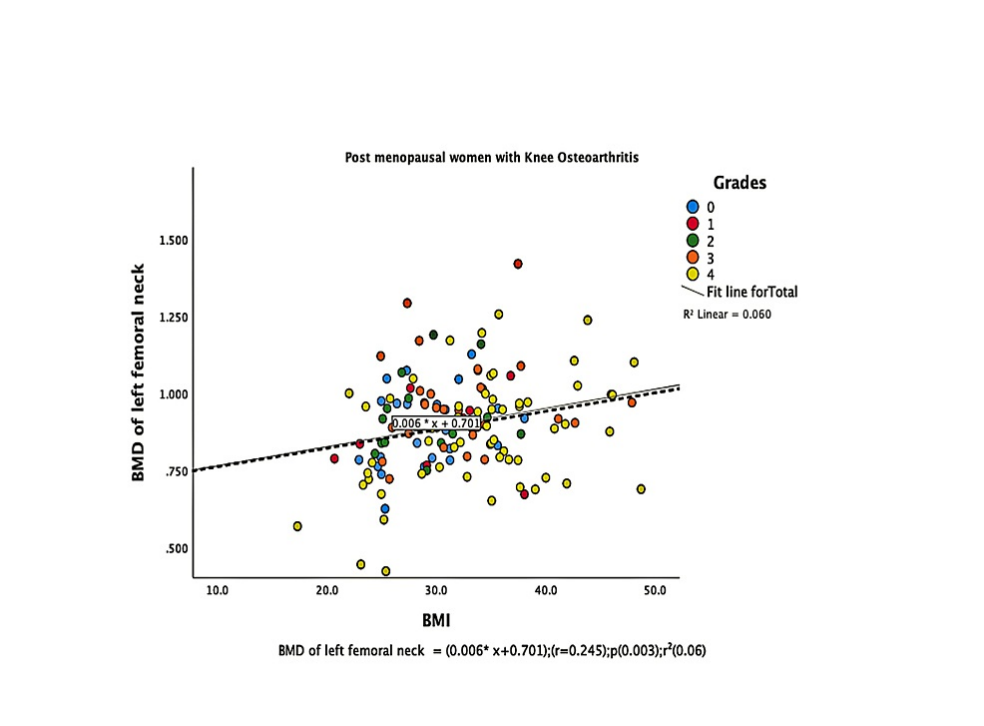
Correlation and regression analyses of relations between BMD of the left femoral neck (Y-axis) and BMI (X-axis) of post-menopausal women with osteoarthritis. BMD: bone mineral density; BMI: body mass index

**Figure 9:**
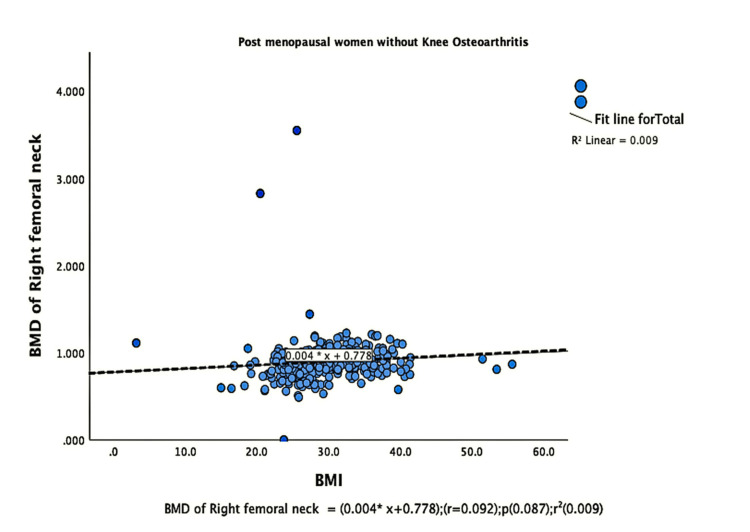
Correlation and regression analyses of relations between BMD of the right femoral neck (Y-axis) and BMI (X-axis) of post-menopausal women without osteoarthritis BMD: bone mineral density; BMI: body mass index

**Figure 10:**
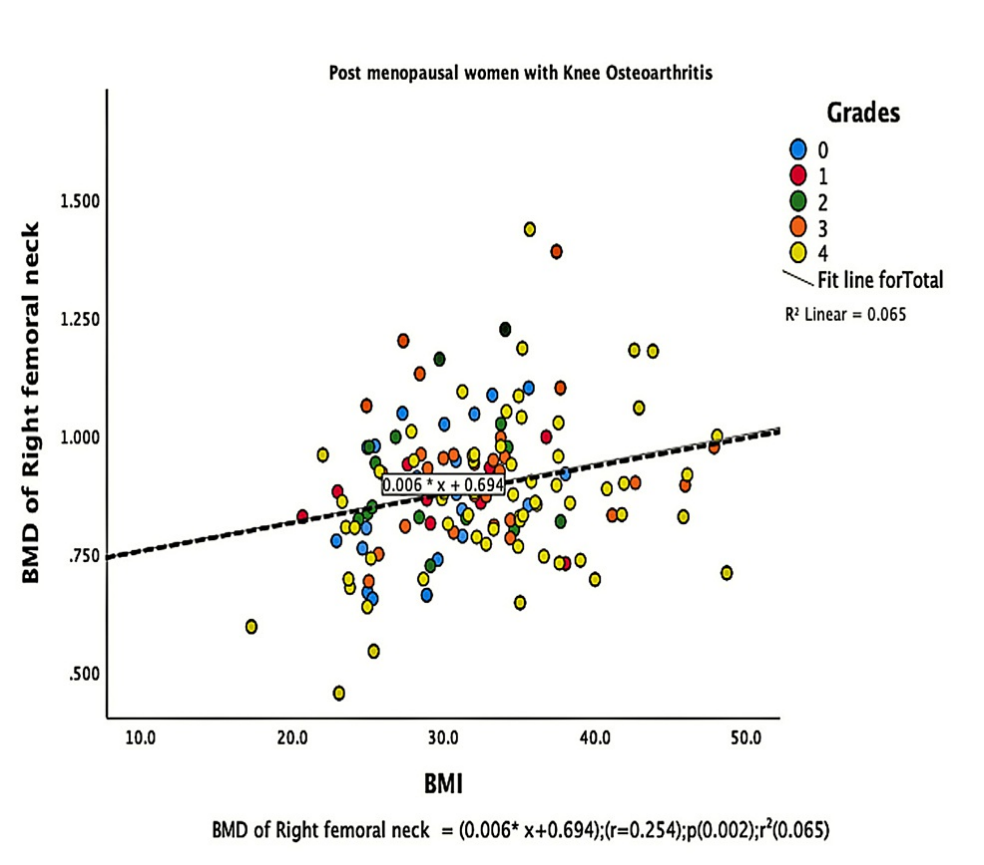
Figure (5B): Correlation and regression analyses of relations between BMD of the right femoral neck (Y-axis) and BMI (X-axis) of post-menopausal women with osteoarthritis. BMD: bone mineral density; BMI: body mass index

**Figure 11:**
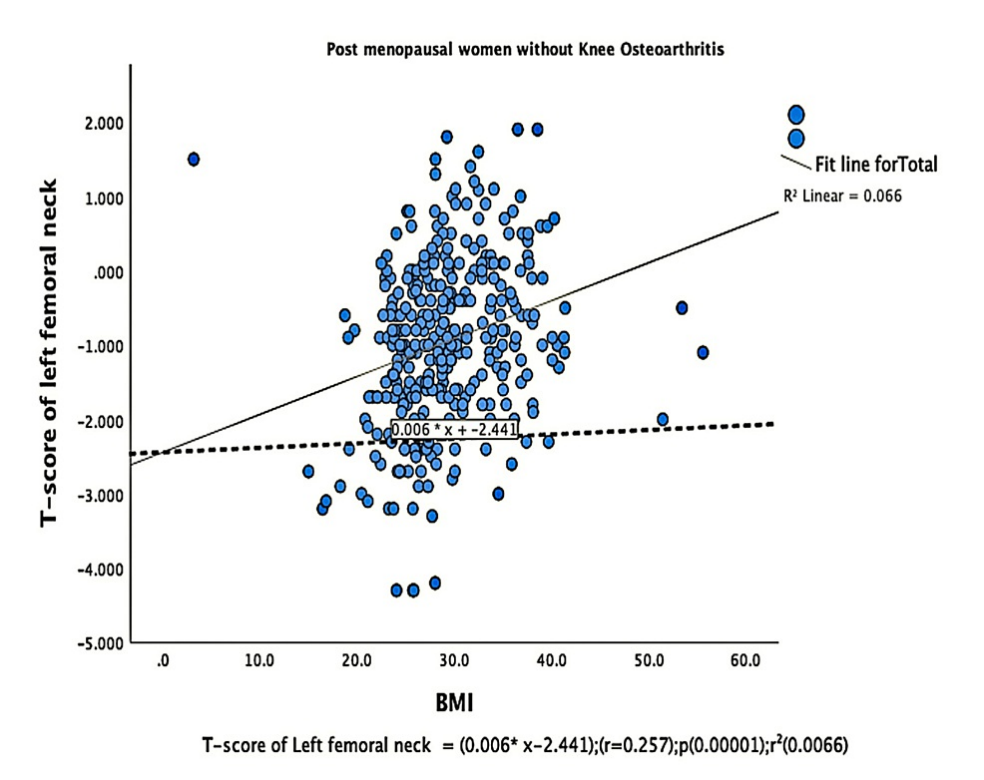
Correlation and regression analyses of relations between the T-score of the left femoral neck (Y-axis) and BMI (X-axis) of post-menopausal women without osteoarthritis BMI: body mass index

**Figure 12:**
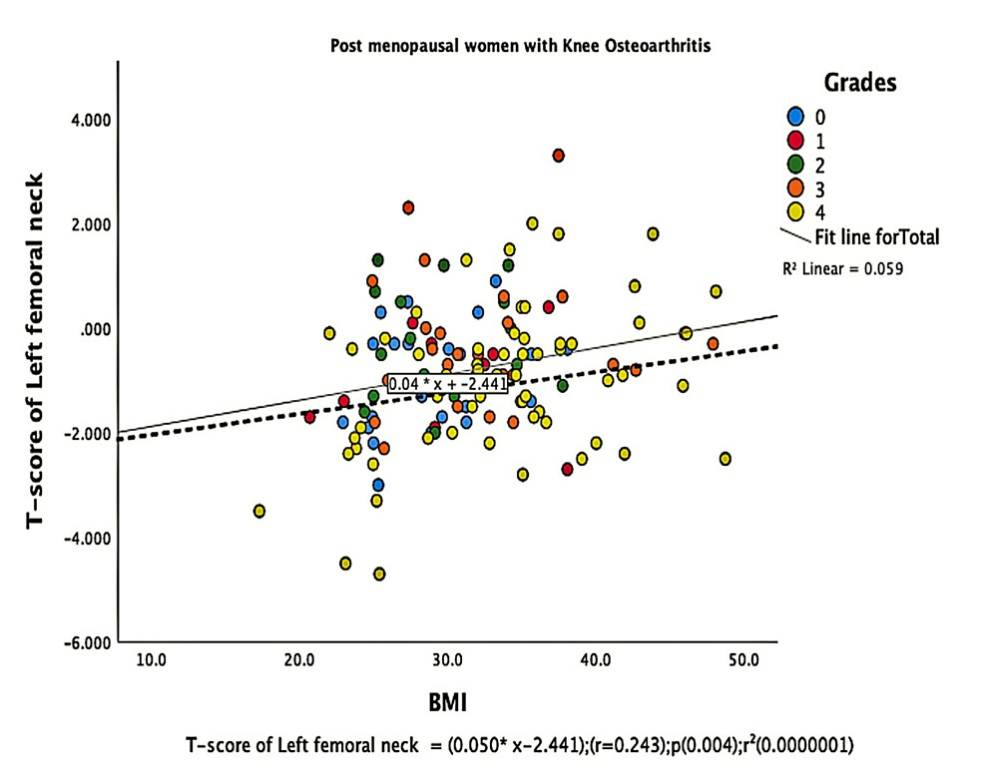
Correlation and regression analyses of relations between the T-score of the left femoral neck (Y-axis) and BMI (X-axis) of post-menopausal women with osteoarthritis.

**Figure 13:**
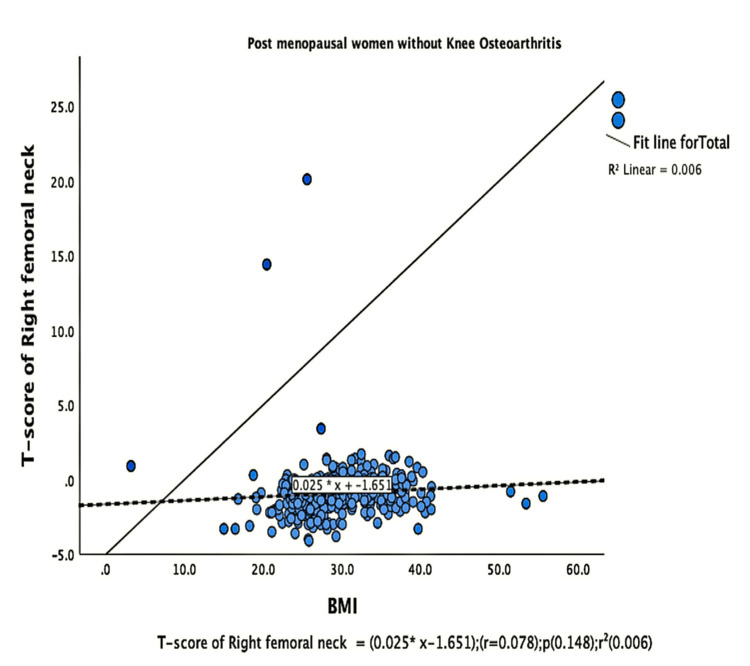
Correlation and regression analyses of relations between the T-score of the right femoral neck (Y-axis) and BMI (X-axis) of post-menopausal women without osteoarthritis

**Figure 14:**
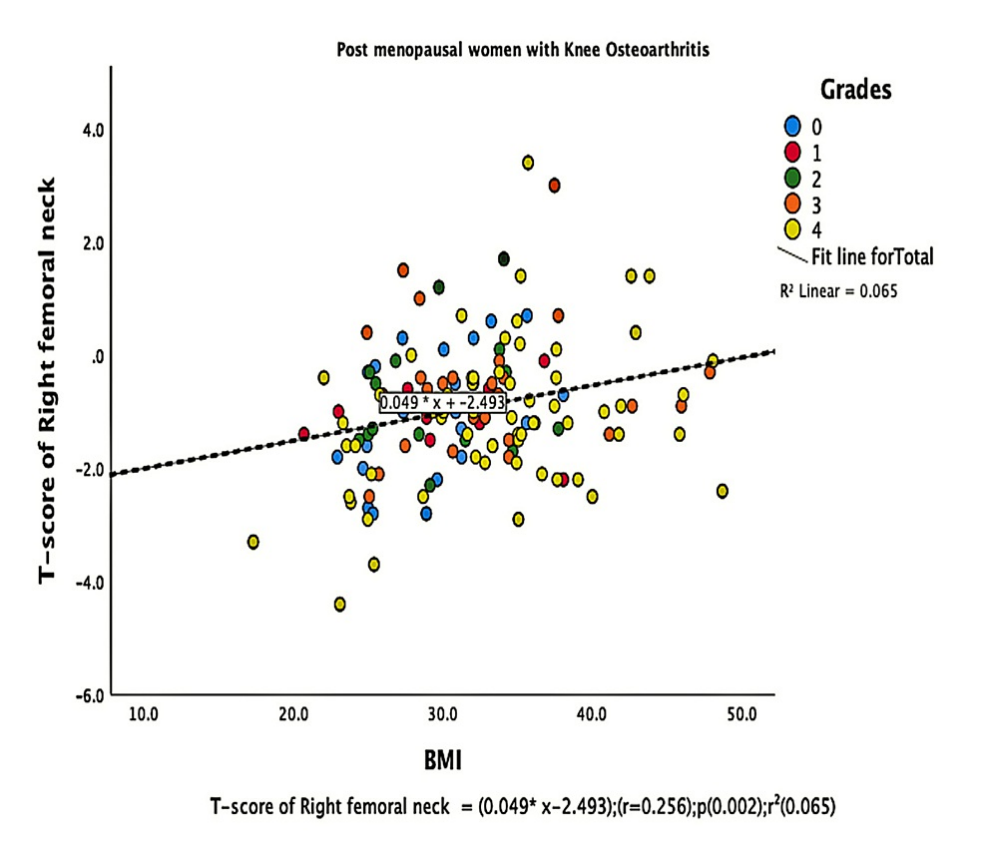
Correlation and regression analyses of relations between the T-score of the right femoral neck (Y-axis) and BMI (X-axis) of post-menopausal women with osteoarthritis

The parameters of bone quality, age, and BMI for all patients were further subjected to correlation and regression analysis with groups I and II of knee OA. In group, I, patients were analyzed according to their grades in the KL classification system. Although the presence of knee OA has a significant relation with BMI and the ages of the patients individually, there was no confounding relationship between age, BMI, and knee OA (Figure 1). There were a positive correlation and significant regression of BMD and T-score of the lumbar spine and the left femoral neck with BMI in both groups of knee OA (Figures 2-8 and Figures 11-12), which means that this correlation is not linked to the presence of knee OA. On the other hand, the BMD and the T-score of the right femoral neck have no significant correlation with BMI in group II. But they have a positive correlation and multiple regression in group I patients with knee OA (figures 9-10, 13-14). This means that this correlation is linked to the presence of knee OA.

## Discussion

The complicated relationship between OA and OP has been debated [3,4]. The inverse relationship between OA and OP has been recognized through surgical experiences and early cross-sectional studies, although the exact pathophysiology remains unclear [8,10-12]. 

The association between OA and OP appears genuine. However, which factors determine the risk of developing either is unclear [4]. Herrero-Beaumont et al. [3] stated that although most clinical data point to an inverse relationship between OA and OP, recent studies indicate that low bone mass in the established OP may also harm joint cartilage. Therefore, they hypothesize that high and low BMD conditions may damage cartilage, and extreme conditions can predispose to OA. However, Hannan et al. [8] concluded that femoral BMD is higher in women with knee osteophytosis but not necessarily associated with joint space narrowing.

The study performed by Hart et al. [10] revealed that small increases in BMD are present in women with knee OA. Their data support the hypothesis that the two conditions of OP and OA are inversely related, though the mechanisms remain uncertain. These findings proposed that some females inherit a bone-forming tendency which may offer small protection against OP but increase the risk of OA. Zhang et al. [13] concluded that higher bone density is associated with an increased prevalence of knee OA. However, their data indicated that greater BMD is strongly associated with a decreased risk of progressive knee OA. 

Bergink et al. [14] found that, despite a higher BMD, subjects with knee OA have an increased risk of fractures, especially vertebral fractures. This relation appears to be driven by osteophytosis. Sowers et al. [7] reported that women with radiographically defined knee OA have greater BMD than women without knee OA. There was less bone turnover among women with hand and knee OA. These findings suggest that OA should also be defined as having a differential response of bone-forming cells with time, at least in pre-and postmenopausal women [7]. On the other hand, Nevitt et al. [11] concluded that femoral neck BMD is significantly higher in the affected hips of older Caucasian women with moderate to severe radiographic findings of hip OA. These findings offer insights into how BMD may affect the course of the most common joint disease and thus may have potential therapeutic suggestions. 

Kim et al. [15] performed a study that included many subjects, in which 5793 people with OA were observed in the Korean National Health and Nutrition Examination Surveys. They demonstrated an independent positive association between the femoral neck and lumbar spine BMDs with knee OA. The same findings were reported by Povoroznyuk et al. [16], where a cross-sectional study was performed in Ukraine that included 359 females aged 50-89 years old. They said that in postmenopausal women, symptomatic knee OA had a significantly higher lumbar spine BMD than women with normal knees.

Contrary to the findings of all these studies mentioned above, the results of the current study didn't show any significant direct correlation or association between OP, BMD, or T-score and knee OA. This could be postulated to some limitations in our study design, or it may be due to population or racial variations. Nevertheless, Atalar et al. [17] reported a similar finding to our results. They performed a cross-sectional study of 95 postmenopausal women aged 49-83 and found no clear correlation between BMD and knee OA. Therefore, the current study's authors believe that the assumption of a simple positive or negative relationship between OP and OA is not appropriate without considering many other potential covariates.

An inverted U-shaped connection between hip BMD and KL grades of knee OA was observed in women but not in men in the study by Hannan et al. [8], which included 932 individuals. Similar but not identical findings were reported by Kim et al. [15]; they displayed an independent negative association as well as a non-linear and site-specific association between OP and the severity of knee OA with KL grades 2, 3, and 4. Our results revealed different findings, where BMD and T-score did not correlate with the presence of knee OA according to the KL grading system. However, the status of bone quality (presence of OP) in patients with knee OA correlated only with KL grade 3.

Our results agree with other studies that reported a significant positive correlation between lumbar spine BMD and BMI [16,18]. Also, Povoroznyuk et al. [16] agree that postmenopausal women with primary knee OA have significantly higher BMI and weight than those with normal knees. Additionally, we observed a significant relationship between patients with OA and their ages. But there was no confounding relationship between age, BMI, and OA. Nevertheless, regression analysis of our data revealed an interesting finding that BMD and T-score of the right femoral neck, together with high BMI, could predict knee OA.

The current study's findings should be considered in a situation with several limitations. First, its nature of being a retrospective, cross-sectional, and single-center study. Second, recruiting patients who are healthy enough to visit the hospital introduces a health-selection bias. Third, using a radiological definition of OA without considering clinical symptoms and signs. Lastly, patient selection ignored those with OA who were not subjected to bone density measurements.

## Conclusions

In conclusion, contradicting many previous studies' hypothesis that radiographically defined knee OA in post-menopausal women has a positive relationship with BMD and an inverse relationship with the presence of OP, we didn't find a clear association between OP or BMD and the company of knee OA. On the other hand, our results concluded that BMD and T-score of the right femoral neck, together with BMI, could predict the occurrence and progression of knee OA. Finally, the authors believe that the hypothesis of a simple positive or negative relationship between the OP and OA is inappropriate without considering many potential confounding variations.
